# Evaluation of Duration of Immunogenicity and Protective Efficacy of Improved Influenza Viral Vector–Based *Brucella abortus* Vaccine Against *Brucella melitensis* Infection in Sheep and Goats

**DOI:** 10.3389/fvets.2020.00058

**Published:** 2020-02-27

**Authors:** Aigerim Mailybayeva, Sholpan Ryskeldinova, Nadezhda Zinina, En-Min Zhou, Gourapura J. Renukaradhya, Kaissar Tabynov

**Affiliations:** ^1^Laboratory of Infectious Disease Prevention, Research Institute for Biological Safety Problems, Gvardeiskiy, Kazakhstan; ^2^Laboratory of Microbiology, Research Institute for Biological Safety Problems, Gvardeiskiy, Kazakhstan; ^3^Department of Preventive Veterinary Medicine, College of Veterinary Medicine, Northwest A&F University, Yangling, China; ^4^Food Animal Health Research Program, Department of Veterinary Preventive Medicine, College of Food Agricultural and Environmental Sciences, The Ohio State University, Wooster, OH, United States; ^5^Biological Safety Department, Kazakh National Agrarian University, Almaty, Kazakhstan; ^6^General Clinical Department, Research Institute of Cardiology and Internal Medicine, Almaty, Kazakhstan

**Keywords:** *Brucella melitensis*, influenza viral vector, vaccine, T-cell–mediated immune response, prolonged efficacy, small ruminants

## Abstract

In this study, we first evaluated the duration of a protective immune response against *Brucella melitensis* infection in non-pregnant sheep and goats immunized with an improved (by vaccine formulation and route of administration) commercial *Brucella abortus* vaccine based on influenza viral vectors expressing *Brucella* immunodominant Omp16, L7/L12, Omp19, or Cu-Zn superoxide dismutase (SOD) proteins (Flu-BA_Omp19-SOD). Sheep and goats in the vaccinated group were immunized thrice concurrently via the subcutaneous and conjunctival routes of administration at an interval of 21 days. Animals in the control group were administered with 20% Montanide Gel01 adjuvant in phosphate-buffered saline in the same way. We showed that the Flu-BA_Omp19-SOD vaccine in sheep and goats induces antigen-specific Th1-biased [immunoglobulin G2a (IgG2a) over IgG1] antibody response and T-cell and interferon γ responses lasting over a period of 1 month post–last vaccination (PLV). The levels of protection against *B. melitensis* 16M infection (vaccination efficacy) in vaccinated sheep for a period of 6 months were 0–20% and in goats 20–40% compared to control challenge group. But the severity of *B. melitensis* 16M infection in the Flu-BA_Omp19-SOD–vaccinated sheep and goats during the entire period of observation revealed the infection index (*P* = 0.001–*P* < 0.0001) and *Brucella* colonization in lymph nodes and organs (*P* = 0.04–*P* < 0.0001) were significantly lower than those in the control group. To conclude, the Flu-BA_Omp19-SOD vaccine using improved formulation and administration method in sheep and goats provides augmented antigen specific humoral and T-cell immune response lasting only for 1 month PLV and partial protection for 6 months against *B. melitensis* 16M infection.

## Introduction

Brucellosis is a chronic infectious disease of animals and humans that induces huge economic losses globally. *Brucella melitensis* is the causative agent of brucellosis in sheep and goats and represents the greatest risk to human health among all known *Brucella* species (1). To control brucellosis in animals, vaccination is one of the most cost-effective measures, which in turn helps in protecting the health of humans in endemic areas (2). This also aids in eradication of the disease among livestock (3). Currently, attenuated *B. melitensis* Rev.1 vaccine is used in sheep and goats (4). Although the Rev.1 vaccine has been found effective, it has several limitations such as it causes abortion in a fraction of vaccinated animals, the vaccine bacteria are virulent to humans, and differentiation of infected from vaccinated animals (DIVA) is a challenge (4, 5). Therefore, development of a safe and effective vaccine to control *B. melitensis* infection in sheep and goats that has DIVA potential is warranted.

Earlier, we developed a novel *Brucella abortus* vaccine based on influenza viral vector (IVV) expressing *Brucella*-immunodominant outer membrane protein (Omp)16 or ribosomal L7/L12 protein (Flu-BA) (6). In January 2019, the Flu-BA vaccine was registered in Kazakhstan (registration certificate no. RK-VP-1-3775-19 dated January 14, 2019) and is currently being used in cattle against *B. abortus* infection. The vaccine response data obtained in cattle (6), as well as information supporting the ability of influenza viruses to infect sheep and goats (7, 8), suggest that vaccines based on IVV can be an effective candidate in small ruminants. It is important to note that the IVV-expressing proteins are immunodominant and common (genetically similar for 95–99%) for *B. melitensis, B. abortus, Brucella suis*, and *Brucella canis* (9–11). Our earlier study with Flu-BA vaccine provided 57.1 and 42.9% efficacy in vaccinated non-pregnant sheep and goats, respectively (12), which prompted us to evaluate the improved Flu-BA vaccine formulation. This formulation had additional IVV-expressing *Brucella* Omp19 and Cu, Zn superoxide dismutase (SOD) proteins, an increased concentration of the adjuvant Montanide Gel01 by 2-fold called Flu-BA_Omp19-SOD, and delivery system (administered the vaccine simultaneously by subcutaneous and conjunctival routes), and the number of doses was increased to three from two and tested in pregnant sheep and goats against *B. melitensis* challenge infection. In pregnant small ruminants, the Flu-BA_Omp19-SOD vaccine was shown to be safe and effective with complete protection (lack of *Brucella* isolation in all animal samples) against *B. melitensis* infection in 66.7% sheep and 55.6% goats (12), whereas the commercial Rev.1 vaccine provides protection against *B. melitensis* infection in 83.3% goats and 100% sheep (12). Because of added benefits of the Flu-BA_Omp19-SOD vaccine, it is considered as a promising candidate. However, it was important to define the extended duration of protective efficacy of the Flu-BA_Omp19-SOD in sheep and goats, which was the objective of this study. The ability of a vaccine to form a long-term protective immune response is one of its most valuable and critical properties, and therefore this research has been decisive in continuing or discontinuing work in this area.

## Materials and Methods

### Bacterial Strains and Biosafety Aspects

The virulent strain *B. melitensis* 16M (obtained from the Research Institute for Biological Safety Problem's collection of microorganisms) was used in this study. The bacterial cells were cultured under aerobic conditions in *Brucella* base agar (Sigma, St. Louis, MO, USA) at 37°C. All experiments with live *Brucella* were performed in level 3 biosafety facilities. Challenged sheep and goats were contained in specialized facilities (biosafety level 3 agricultural).

### Vaccine Preparation

Vaccines were prepared from the four IVV subtypes A/H5N1 expressing the *Brucella* L7/L12 or Omp16 or Omp19 or Cu-Zn SOD (SOD) proteins from the open reading frame of the NS1 gene (Flu-NS1-124-L7/L12-H5N1, Flu-NS1-124-Omp16-H5N1, Flu-NS1-124-Omp19-H5N1, Flu-NS1-124-SOD-H5N1), which were generated previously (13). A detailed description of the vaccine preparation procedure is described previously (14). An improved tetravalent vaccine formulation expressing the *Brucella* L7/L12, Omp16, Omp19, SOD proteins was provisionally referred to as the Flu-BA_Omp19-SOD. The lyophilized vaccine before administration was resuspended (2.5 mL per ampule) in a 20% solution of the adjuvant Montanide Gel01 (Seppic, Puteaux, France) in phosphate-buffered saline (PBS).

### Vaccination, Study Design, and Sampling

**Figure 1:**
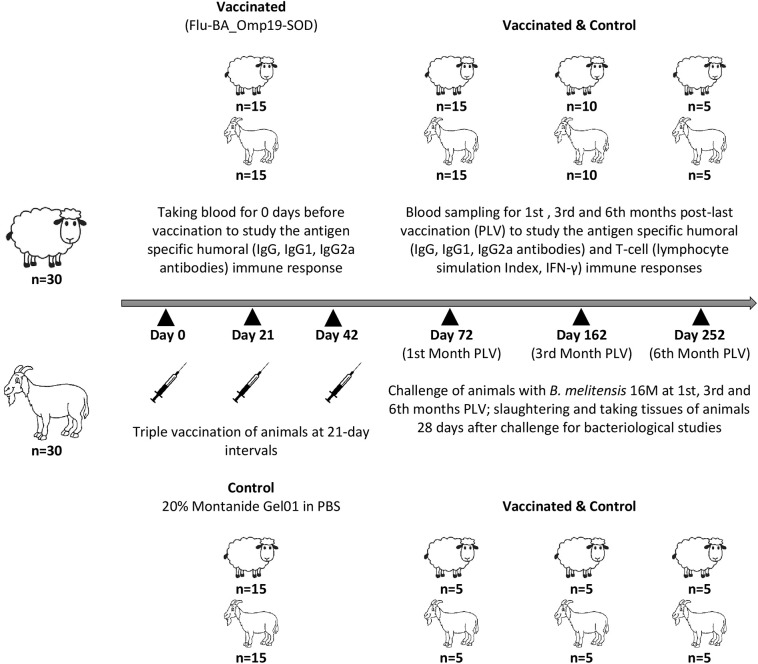
Experimental design.

A total of 30 Degeresskaya semifine meat and wool purpose breed of sheep and 30 Gorno-Altaisk breed of goats aged 6–18 months from an officially brucellosis-free flocks were used in this study. All animals were non-pregnant females. Further, two groups were formed from each animal species by randomization method: the Flu-BA_Omp19-SOD–vaccinated and control groups (*n* = 15/group). Sheep and goats in the vaccinated group were immunized thrice concurrently via the subcutaneous (2.0 mL in the axillary region) and conjunctival (0.25 mL to each eye) routes of administration at an interval of 21 days with the vaccine dose 7.0 log_10_ EID_50_/animal. Animals in the control group were administered with 20% Montanide Gel01 adjuvant in PBS in the same way. The antigen-specific enzyme-linked immunosorbent assay (ELISA) antibodies (total IgG, IgG2a, and IgG1), lymphocytes stimulation index (SI), and interferon γ (IFN-γ) production in animals both before and at the first (*n* = 15/group), third (*n* = 10/group), and sixth (*n* = 5/group) months post–last (third dose) vaccination (PLV) in serum and whole-blood samples were performed. At the first (*n* = 5/group), third (*n* = 5/group), and sixth (*n* = 5/group) months PLV, sheep and goats from the vaccinated and control groups were challenged with a virulent strain of *B. melitensis* 16M at a dose of 10^6^ colony-forming units (CFU)/animal subcutaneously (axillary region right side). At 28 days after challenge, the animals were euthanized and slaughtered aseptically for sampling. Schematic representation of the study design is shown in Figure 1.

### Antibody Response

This study was conducted in full accordance with our previously published work (14). Briefly, 96-well microtiter plates (Nunc, Roskilde, Denmark) were coated overnight with pre-titrated mixture of *Brucella* L7/L12, Omp16, Omp19, or SOD proteins (each at 2 μg/mL) in PBS, blocked for 1 h using PBS containing 1% ovalbumin (PBS-OVA; 200 μL/well), and washed with PBS containing 0.05% Tween-20 (PBS/Tw). Serial 2-fold dilutions of the serum samples in PBS/OVA were added (100 μL/well) to the plates and incubated for 1 h at room temperature. Donkey antiruminant IgG horseradish peroxidase conjugate (Sigma) and monoclonal antibodies specific for sheep IgG1 and IgG2 (Novus Biologicals, Littleton, CO, USA) were used for detection of total and isotype-specific antibodies. After 90-min incubation at 37°C, plates were washed, and the specific reactivity was determined by addition of an enzyme substrate ABTS [2,2_azinobis(3-ethylbenzthiazolinesulfonic acid)] diammonium (Moss, Inc., Pasadena, CA, USA) at 100 mL/well. The absorbance values were measured at 415 nm. Antibody levels were expressed as the arithmetic mean ± standard errors of the optical density (OD) value obtained for sheep and goats samples included in each group.

### Preparation of Peripheral Blood Mononuclear Cell for Lymphocyte Proliferation Assay

This work was carried out in accordance with Mailybayeva et al. (14). Briefly, peripheral blood mononuclear cells (PBMCs) were isolated by density gradient centrifugation using the Ficoll–sodium diatrizoate gradient (DNA-Technology, Moscow, Russia). Cells number was adjusted to 10^7^ viable cells per mL determined by trypan blue dye exclusion, and 50 μL of each cell suspension (containing 5 × 10^5^ cells) was added to eight separate flat-bottomed wells of 96-well microtiter plates already plated with 100 μL of RPMI-1640 medium only or RPMI-1640 medium containing 8.0 μg of purified *Brucella* proteins L7/L12, Omp16, Omp19, or SOD per well. The cell cultures were incubated for 7 days at 37°C under 5% CO_2_. After incubation, the cells were pulsed with 1.0 μCi of [^3^H] thymidine per well for 18 h. Cells were harvested onto glass filter mats and counted for radioactivity in a liquid scintillation counter. Cell proliferation results were converted to SI [counts per minute (cpm) of wells containing antigens/cpm in the absence of antigens] for comparison.

### Cytokine IFN-γ Production

This study was also conducted in accordance with Mailybayeva et al. (14). Briefly, PBMCs from each animal were adjusted to 10^7^ viable cells per mL as described in the previous paragraph. Aliquots (50 μL) of each cell suspension containing 5 × 10^5^ cells were plated and stimulated with *Brucella* proteins L7/L12, Omp16, Omp19, or SOD as described above. Cell cultures were incubated at 37°C under 5% CO_2_, and the supernatants were harvested 72 h later and assayed for IFN-γ using a commercial ELISA kit (RayBio1Bovine IFN-γ ELISA Kit; RayBiotech, Inc., Norcross, GA, USA). This kit has been shown to cross-react with IFN-γ of sheep and goats (15). Antigen-specific IFN-γ production was determined for each individual animal by subtracting the background concentration of IFN-γ in wells without antigen from the IFN-γ concentration in wells with antigen.

### Assessment Protective Efficacy of the Vaccine in Sheep and Goats

Samples of lymph nodes (submandibular, retropharyngeal, right subscapular, left subscapular, mediastinal, bronchial, portal, para-aortic, pelvic, mesenteric, udder) and parenchymal organs (liver, kidney, spleen, and bone marrow) were taken from slaughtered animals. In total, 15 organs were sampled from each animal. Bacteriological studies and evaluation of results were carried out as described in the previous study (12). Briefly, the tissue homogenates in 0.1% Triton–PBS after 10-fold serial dilutions were plated onto *Brucella* base agar plates and incubated at 37°C for 2 weeks, and the growth of bacterial colonies was counted periodically during this time. The concentrations of bacteria [colony-forming units (CFU)/g of tissue] in the tissue samples were determined by performing standard plate counts. An animal was considered infected if a *Brucella* colony was detected from the culture of one or more organs. The results of the bacteriological study were evaluated by the following three parameters: (A) vaccination efficacy or number of animals (expressed in %) with complete protection against infection (lack of *Brucella* isolation in all animal samples); (B) generalization of the infectious process or infection index (number of organs and lymph nodes of animals in which *Brucella* are isolated; the arithmetic mean was given); (C) intensity/severity of the infectious process or the degree of *Brucella* colonization from samples of lymph nodes and organs (expressed as log_10_ CFU/g of tissue).

### Statistical Analysis

Differences in protective efficacy (complete protection vs. infection in animals) between groups were compared by one-sided Fisher exact test at a significance level of α < 0.05. The significance in antibody responses (IgG, IgG1, and IgG2a), SI, concentration of IFN-γ, the index of infection, and colonization of *Brucella* in tissues between groups was analyzed using two-way analysis of variance followed by Sidak's multiple-comparisons test. *P* < 0.05 was considered significant. Means are reported with standard errors (SEM). Statistical analysis of all experimental data was performed with GraphPad Prism Software version 6.0 (GraphPad Software Inc., La Jolla, CA, USA).

## Results

### Antibody Response to *Brucella* Proteins in Animals

**Figure 2:**
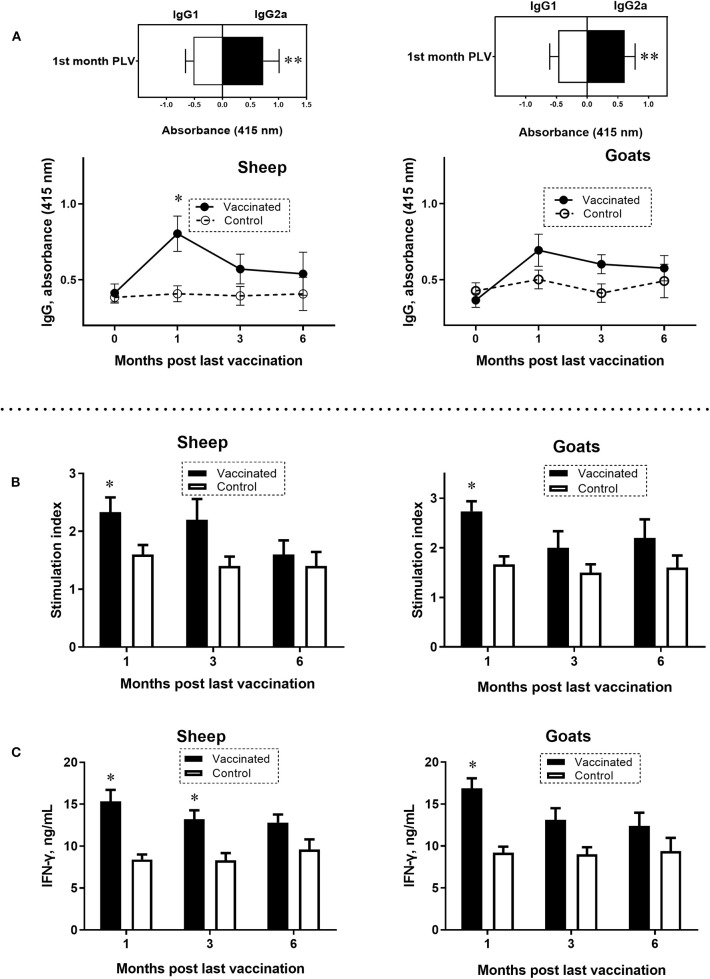
Antigen-specific enzyme-linked immunosorbent assay (ELISA) IgG, IgG1, and IgG2a antibody responses **(A)** and lymphocyte stimulation index **(B)** and interferon γ (IFN-γ) production **(C)** in peripheral blood mononuclear cells (PBMCs) of sheep and goats at 0 or first, third, and sixth month PLV. A mixture of *Brucella* L7/L12, Omp16, Omp19, and superoxide dismutase proteins was used to simulate PBMCs and in ELISA. Sheep and goats were vaccinated thrice concurrently via the subcutaneous and conjunctival route. Animals in the control group received only the adjuvant in phosphate-buffered saline. Statistical analysis was performed using two-way analysis of variance followed by Sidak's multiple-comparisons test. ELISA antibody levels were presented as OD ± standard error. Cell proliferation results were converted to stimulation index [counts per minute (cpm) of wells containing antigens/cpm in the absence of antigens] for comparison. Antigen-specific IFN-γ production was determined for each individual animal by subtracting the background concentration of IFN-γ in wells without antigen from the IFN-γ concentration in wells with antigen. *P = 0.0007 vaccine group vs. control; **P = 0.014–0.02 IgG2a vs. IgG1 at the first month PLV. Data of lymphocyte stimulation index and levels of IFN-γ are presented as mean ± standard error; *P = 0.047–*P* < 0.0001 vaccine vs. control group.

In the serum of vaccinated sheep and goats, IgG antibody response to a mixture of *Brucella* L7/L12, Omp16, Omp19, and SOD proteins was at peak levels after 1 month PLV. In sheep, the antibody levels were significantly higher (*P* = 0.0007) compared to control (Figure 2A). Immunoglobulin G antibody responses in goats did not differ at any of the sampling times. Analysis of isotype specific antibodies in sheep and goats at the first month PLV revealed significantly (*P* = 0.014–0.02) higher IgG2a over IgG1 (Figure 2A), indicating the Th1-polarized immune response. At the third and sixth months PLV in vaccinated sheep and goats, reduced production of antibodies was observed, and the data were not statistically significant (*P* = 0.33–0.97) compared to control animals values.

### Lymphocyte Proliferation Responses and IFN-γ Production After Vaccination

At the first month PLV, analysis of antigen (*Brucella* L7/L12, Omp16, Omp19, and SOD proteins)–specific lymphocyte SI values in PBMC (sheep 2.33 ± 0.25; goats 2.73 ± 0.20; *P* = 0.047–0.0009; Figure 2B) and production of IFN-γ (sheep 15.33±1.36 ng/mL; goats 16.86±1.20 ng/mL; *P* < 0.0001; Figure 2C) were significantly higher compared to control animals, indicating the robust T-cell response to vaccine. While at the third and sixth months PLV, although the lymphocyte SI (sheep by 12.5–36.3%, goats by 25.0–27.2% higher) and IFN-γ production (sheep by 27.2–35.1%, goats by 27.4–28.2% higher) values were higher than those in the control animals, the overall T-cell response in vaccinated sheep and goats was not statistically significant (SI, sheep 1.6 ± 0.24–2.2 ± 0.35, goats 2.0 ± 0.33–2.2 ± 0.37, *P* = 0.09–0.97; IFN-γ, sheep 12.8 ± 0.97–13.2 ± 1.07 ng/mL; goats 12.4 ± 1.56–13.1 ± 1.4 ng/mL; *P* = 0.052–0.5).

### Vaccine Protection in Sheep and Goats Against *B. melitensis* Infection

**Figure 3:**
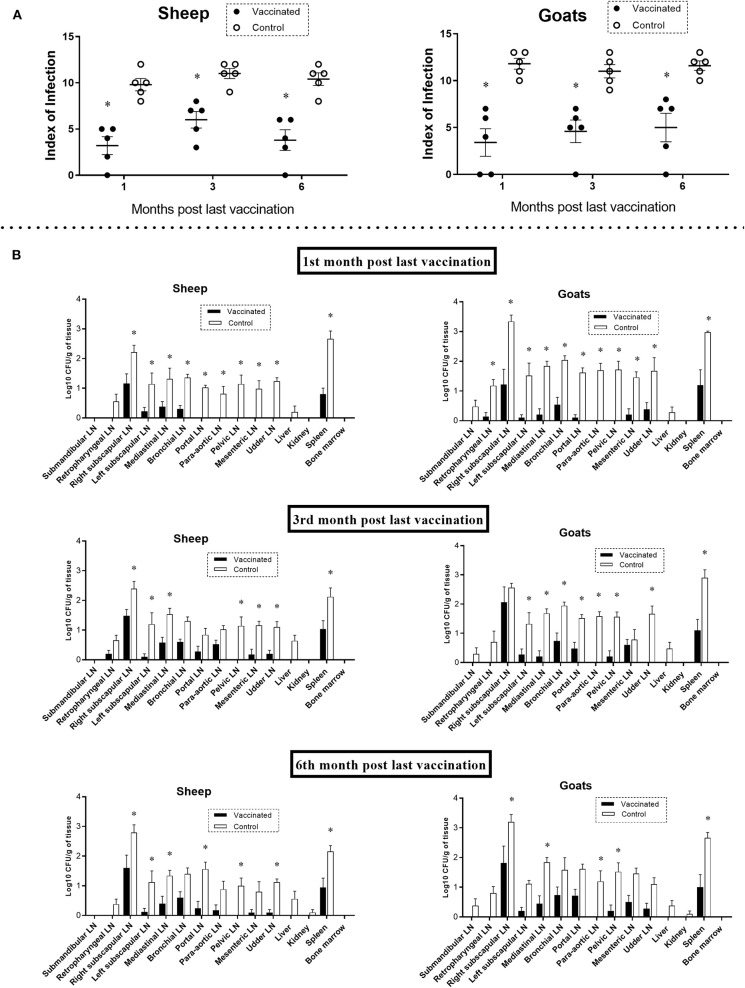
Index of *Brucella* infection **(A)** and *Brucella melitensis* colonization in tissues **(B)** of sheep and goats challenged with *B. melitensis* 16M at first, third, and sixth month post–last vaccination. Sheep and goats in the vaccinated group were immunized thrice concurrently via the subcutaneous and conjunctival routes. Animals in the control group were administered with adjuvant in phosphate-buffered saline. Animals were challenged with the virulent strain of *B. melitensis* 16M at a dose of 10^6^ CFU/animal via subcutaneous route. The bacteriological examination was assessed by the index of infection in animals (number of organs and lymph nodes from which *Brucella* was isolated in each animal; the arithmetic mean ± standard error was given) and colonization of *Brucella* in tissues (the data were given as log_10_ CFU/g). Statistical analysis was performed using two-way analysis of variance followed by Sidak's multiple-comparisons test. The data of index of infection and colonization of *B. melitensis* in tissues are presented as mean ± standard error; *P = 0.04–*P* < 0.0001 vaccine vs. control group. LN, lymph node.

The duration of the Flu-BA_Omp19-SOD vaccine protective efficacy in sheep and goats against *B. melitensis* 16M infection was assessed using parameters such as vaccination efficacy (level of full protection against infection expressed in %), infection index, and *Brucella* colonization in tissues and organs. Our results showed (Table 1) that the levels of protection against *B. melitensis* 16M infection (vaccination efficacy) in vaccinated sheep were 0–20% and 20–40% in goats compared to the control challenge group (where the animal infection rate was 100%), and the data were not statistically significant (α = 0.22–0.5). But the severity of *B. melitensis* 16M infection in the Flu-BA_Omp19-SOD–vaccinated sheep and goats during the entire period (for up to 6 months PLV) of observation revealed the infection index (Figure 3A: 3.2 ± 0.9–6.0 ± 0.8; *P* = 0.001–*P* < 0.0001) and *Brucella* colonization in lymph nodes and organs (Figure 3B: 0.1 ± 0.1–2.0 ± 0.5 log_10_ CFU/g of tissue; *P* = 0.04–*P* < 0.0001) were significantly lower than those in the control group (infection index 9.8 ± 0.6–11.8 ± 0.5; *Brucella* colonization 0.1 ± 0.1–3.3 ± 0.2 log_10_ CFU/g of tissue). The maximum protection in vaccinated sheep and goats against *B. melitensis* 16M infection was in the first month PLV. At 1 month PLV compared to the third and sixth months PLV, we observed better vaccination efficacy (20–40% vs. 0–20%), a lower index of infection (or number of *Brucella* isolated organs and lymph nodes in each animal: 3.2–3.4 vs. 3.8–6.0), and degree of *Brucella* colonization in tissues [the number of lymph nodes and organs where *Brucella* colonization values were significantly less (*P* < 0.05) than those of the control group: 10–11 vs. 5–8], indicating that the protective efficacy was reduced after 3 and 6 months PLV.

**Table 1:** Rates of infection in the sheep and goats after challenge with the virulent strain *Brucella melitensis* 16M.

**Post–last vaccination period**	**Group**	**Isolation of *B. melitensis* in sheep, *n* (%)**	**Total**	**Isolation of *B. melitensis* in goats, *n* (%)**	**Total**
1st month	Vaccinated	4 (80)	5	3 (60)	5
	Control	5 (100)	5	5 (100)	5
3rd month	Vaccinated	5 (100)	5	4 (80)	5
	Control	5 (100)	5	5 (100)	5
6th month	Vaccinated	4 (80)	5	4 (80)	5
	Control	5 (100)	5	5 (100)	5

## Discussion

The Flu-BA vaccine was commercialized to use in cattle in Kazakhstan. Earlier, we tested this vaccine's efficacy after improving the formulation (Flu-BA_Omp19-SOD) and delivery system in pregnant sheep and goats and observed promising safety and efficacy (14). In this study, we for the first time evaluated the duration of protective responses in non-pregnant sheep and goats induced by the candidate vaccine. Earlier, we conducted short-term pilot studies in both non-pregnant and pregnant small ruminants. In this study, the protracted duration of effectiveness of the vaccine for up to 6 months PLV was evaluated.

The protective efficacy of the Flu-BA_Omp19-SOD vaccine in non-pregnant small ruminants at the first month PLV against *B. melitensis* infection was 20% in sheep and 40% in goats, whereas in pregnant sheep and goats, it was 66.7 and 55.6%, respectively (14), wherein similar vaccine formulation and immunization regimen were followed. The severity of *B. melitensis* infection in vaccinated sheep and goats in this study was measured by infection index (3–3.4 times lower than control) and *Brucella* colonization in tissues (lower by 131 times than control), which was inferior to those in pregnant animals (infection index 4.5–9.6, *Brucella* colonization >200 times than control) (14). However, the antigen-specific humoral and especially T-cell responses, which play an important role in the antibrucellosis immunity (16, 17), were comparable in both those studies. The Flu-BA_Omp19-SOD vaccine–induced antigen-specific IgG antibodies (IgG2a vs. IgG1), lymphocyte SI, and IFN-γ production in sheep and goats at the first month PLV were less (SI: 2.3–2.7 vs. 3.1–3.7; IFN-γ production: 15.3–16.8 vs. 19.1–19.4 ng/mL) than those in published similar pregnant small ruminant study (14). We partly attribute the lower protective efficacy observed in this study to a wide range in age differences (6–18 months) in experimental animals used and small numbers included in each group (*n* = 5) compared to the findings in an earlier study (14). This assumption is consistent with previous work (18), wherein responses by PBMCs measured by two different assays between different sheep and within sheep over different sample time points varied substantially in terms of cytokine production and proliferation. In all previous experiments, animals used were more age-homogeneous, as well as in relatively larger numbers of younger animals (3–4 months, *n* = 7/group or 9–10 months, *n* = 9/group) (12, 14). Unfortunately, it has not been possible to reliably determine the effectiveness of the Flu-BA_Omp19-SOD vaccine on the adult immunized sheep and goats in this study. In any case, in this study, we used a sufficient number of animals in each group, which allowed us to obtain statistically reliable data.

At the third and sixth months PLV, we observed not only reduced Flu-BA_Omp19-SOD vaccine efficacy but also decreased humoral and T-cell responses. However, it is important to note that the severity of *B. melitensis* 16M infection in vaccinated sheep and goats at the indicated time of observation, estimated by the infection index and the degree of *Brucella* colonization from tissues, was significantly lower than in the control group. Our results demonstrated that a partial protection was induced by the Flu-BA_Omp19-SOD vaccine in sheep and goats for at least 6 months PLV.

In comparison of our results on the duration of protective responses of the Flu-BA_Omp19-SOD vaccine in sheep and goats with the available vaccines (19, 20), it is clear that our candidate vaccine provided that it is insufficient to provide complete protection against infection. For example, the commercial vaccine (*B. melitensis* Rev.1) provides more than 80% full protection in vaccinated small ruminants for ~2–5 years (19). The Flu-BA vaccine after prime-boost immunization provides at least 12 months' antigen-specific T-cell immune response and protection in 57% of cattle against *B. abortus* 544 infection (20). The apparent difference in the duration of the protective antibrucellosis immune response in cattle (20) and small ruminants vaccinated with the same vaccine type indicates that IVV-based technology is most appropriate for cattle, and less so for sheep and goats. This can be explained by the fact that cattle are more sensitive to influenza A viruses (7, 8), and consequently, our IVVs effectively express *Brucella* proteins and induce a more pronounced immunity. Comparative analysis of all these data with the Flu-BA_Omp19-SOD vaccine (12, 14) indicates that it does not meet the important requirement of prolonged complete protective immunity in sheep and goats, indicating the need of improvements in the vaccine formulation, dosage, and immunization regimen. Further, it is important to note that the present study was performed using non-pregnant small ruminants, which are less sensitive to brucellosis infection (attributed to the presence of erythritol in the pregnant ruminant's placenta, an important growth factor of *Brucella*) (21, 22), and therefore expected higher vaccine efficacy than in pregnant animals, but the result was opposite. However, we still need to perform duration of protective immune responses to the Flu-BA_Omp19-SOD vaccine in pregnant sheep and goats. Consistent with our vaccine results, a similar attempt to use the commercial *B. abortus* vaccine RB51 in small ruminants against *B. melitensis* infection was unsuccessful (23).

To conclude, the Flu-BA_Omp19-SOD vaccine using improved formulation and administration method in sheep and goats provides augmented antigen-specific humoral and T-cell immune response lasting for only 1 month PLV and partial protection for 6 months against *B. melitensis* 16M infection.

## Data Availability Statement

All datasets generated for this study are included in the article/supplementary material.

## Ethics Statement

This study was carried out in compliance with national and international laws and guidelines on animal handling. The protocol was approved by the Committee on the Ethics of Animal Experiments of the Research Institute for Biological Safety Problems of the Science Committee of the Ministry of Education and Science of the Republic of Kazakhstan. Animals were euthanized using sodium pentobarbital anesthetic and all recommended efforts were taken to minimize suffering.

## Author Contributions

KT, AM, SR, and NZ: conception and design of the study, or acquisition of data, or analysis and interpretation of data. KT: drafting the article or revising it critically for important intellectual content. KT, GR, and E-MZ: final approval of the version to be submitted.

### Conflict of Interest

The authors declare that the research was conducted in the absence of any commercial or financial relationships that could be construed as a potential conflict of interest.
